# Platyrrhine color signals: New horizons to pursue

**DOI:** 10.1002/evan.21798

**Published:** 2019-10-14

**Authors:** Laís A. A. Moreira, Gwen Duytschaever, James P. Higham, Amanda D. Melin

**Affiliations:** ^1^ Department of Anthropology & Archaeology University of Calgary Calgary Alberta Canada; ^2^ Department of Anthropology New York University New York; ^3^ Department of Medical Genetics University of Calgary Calgary Alberta Canada; ^4^ Alberta Children's Hospital Research Institute Calgary Alberta Canada

**Keywords:** chroma, color vision, communication, luminance, New World monkeys, skin

## Abstract

Like catarrhines, some platyrrhines show exposed and reddish skin, raising the possibility that reddish signals have evolved convergently. This variation in skin exposure and color combined with sex‐linked polymorphic color vision in platyrrhines presents a unique, and yet underexplored, opportunity to investigate the relative importance of chromatic versus achromatic signals, the influence of color perception on signal evolution, and to understand primate communication broadly. By coding the facial skin exposure and color of 96 platyrrhines, 28 catarrhines, 7 strepsirrhines, 1 tarsiiform, and 13 nonprimates, and by simulating the ancestral character states for these traits, we provide the first analysis of the distribution and evolution of facial skin exposure and color in platyrrhini. We highlight ways in which studying the presence and use of color signals by platyrrhines and other primates will enhance our understanding of the evolution of color signals, and the forces shaping color vision.

## 1 INTRODUCTION

Colorful visual signals, their evolution, and their role in communication within and between species have captivated biologists for centuries.^1^, ^2^ While bold and high‐contrasting colors and patterns are seen throughout diverse animal taxa (e.g. velvet ants [*Dasymutilla occidentalis*],^3^ strawberry poison‐dart frog [*Oophaga pumilio*],^4^ and glass eye squirrel fish *[Heteropriacanthus cruentatus*]^5^), these signals are typically aposematic in function.^6^ Yet some taxa make extensive use of color for intraspecific social and sexual signaling.^7^, ^8^ Arguably the most visually striking of all taxa, the birds are well known for using complex color patterns in their plumage—or more rarely, skin—combined with other signals in conspicuous multimodal displays that often seem to be evaluated by conspecifics and used in female mate choice.^9^, ^10^, ^11^, ^12^, ^13^ While most mammals are typically dull in coloration, nonhuman primates (hereafter, primates) are one obvious exception. Unlike other large mammals, primates often exhibit conspicuous color patterns, most especially on their faces and rumps. A role of colorful signals among primates is perhaps unsurprising, given that visual communication is extensively used in this clade, which exhibit large eyes, high visual acuity, and a heavy reliance on the visual sense for daily activities.^14^, ^15^ Primate skin coloration appears to be of exceptional comparative interest. Unlike in birds, in which coloration is often a product of female mate choice for male ornamentation, in primates, colors seem more commonly linked to male–male competition, and status signaling (e.g. vervet monkeys^16^; geladas^17^; drills^18^; mandrills^19^). Nonetheless, a number of species appear to show traits that are primarily under intrasexual selection and associated with male–male signaling, but that might be secondarily under intersexual selection as mate choice ornaments (e.g., mandrills^19^, ^20^), or conversely, primarily under intersexual selection as mate choice ornaments, but secondarily under intrasexual selection and associated with male–male signaling (e.g., male rhesus macaques^21^, ^22^). Together, this provides a unique and exciting opportunity to investigate total sexual selection—how multiple mechanisms of sexual selection are acting in concert or antagonistically on the same trait.^23^ Additionally, unlike clades like birds, in which all diurnal species are thought to be tetrachromatic, primates exhibit great intraorder diversity in color perception.^24^ Primates also exhibit a stunning range of social organizations, and mating systems, including monogamy, polygyny, polyandry, and polygynandry. The latter includes extreme interspecific variation in the distribution of matings and genetic reproduction.^25^

Understanding the evolutionary selective pressures acting on color variation of the faces, genitals, hindquarters, and elsewhere, within and between primate species has long been of interest to evolutionary biologists and anthropologists, dating back to Darwin.^1^, ^26^ However, with few recent exceptions,^27^, ^28^, ^29^ the vast majority of research has focused on one particular taxonomic lineage, the African and Asian monkeys (part of the Parvorder Catarrhini).^30^ A more comprehensive understanding of primate color variation—as well as a broader intraorder comparative framework for interpreting the radiation of catarrhine color patterns, is currently lacking. Our aim here is to highlight the potential of the other major diurnal radiation of haplorhine primates, the platyrrhines (monkeys inhabiting Mexico, Central, and South America), for new studies of visual communication, which are likely to offer new insights into our understanding of sexual selection and communication in primates.

## 2 SKIN COLOR AND EXPOSURE IN PRIMATES: ROLES IN VISUAL COMMUNICATION

Catarrhine primates are well known to possess large patches of exposed skin on their faces and hindquarters, and to possess skin color that varies in response to fertility, pregnancy, and other reproductive phases, as well as social status.^31^ In addition to chroma (the hue and saturation components of color), patterns of luminance variation is a frequent but often underemphasized component of color signaling in catarrhines.^32^, ^33^ Chromatic and achromatic components of signals seem to have different mechanisms of inheritance^34^ but often covary.^35^ For example, red male rhesus macaques (*Macaca mulatta*) receive more sexual solicitations by more females than pale pink males, suggesting the potential importance of both chroma and luminance.^21^, ^36^ A now impressively large body of work has focused on the reddish and other colorful signals of African and Asian primates, and their roles in socio‐sexual communication (Table 1).

**Table 1:** Studies conducted on primate coloration in relation to social and sexual selection in Catarrhini (African and Asian) and Platyrrhini (Mexican, Central, and South American) monkeys

World region	Species studied	Conclusions	Study methods	Reference
Africa and Asia	Rhesus macaque (*Macaca mulatta*)	Females show a peak in skin coloration during the ovarian cycle	Free‐ranging and captive; color charts; color scores	37
		Females exhibit preferences for the red version of male faces	Free‐ranging; behavior experiment with color‐manipulated digital images of faces	38
		Males display longer gaze durations in response to reddened versions of females’ hindquarters, but not to reddened versions of faces	Free‐ranging; behavior experiment with color‐manipulated digital images of faces	39
		Females had longer gaze durations toward red in comparison to nonred females faces and hindquarters Pregnancy coloration might be an attention‐grabbing stimulus to males	Free‐ranging; behavior experiment with color‐manipulated digital images of faces and hindquarters Free‐ranging; behavior experiment with color‐manipulated digital images of faces	40 41
		Facial skin color covaries with the timing of the fertile phase of the menstrual cycle	Free‐ranging; digital photography; objective measure of color	42
		Facial skin luminance covaries with the timing of the fertile phase of the menstrual cycle	Free‐ranging; digital photography; species‐specific visual models	43
		Males distinguish ovulatory from preovulatory faces, but familiarity seems to be important to perceive signals related to reproductive status	Free‐ranging; behavior experiment with printed color‐calibrated images of faces	44
		No evidence that skin coloration is related to male dominance rank or used in female mate choice	Free‐ranging; digital photography; species‐specific visual models	35
		Dark red males receive more sexual solicitations, by more females, than pale pink ones	Free‐ranging; digital photography; species‐specific visual models and behavior assessment	21
		Adult females and males looked longer at dark male faces compared with pale pink ones	Free‐ranging; behavior experiment with printed color‐calibrated images of faces	36
		Trichromacy confers a better ability to detect meaningful variation in primate face coloration than dichromacy does	Digital images of free‐ranging rhesus macaques; species‐specific visual models	45
	Japanese macaque (*Macaca fuscata*)	Degree of facial redness and occurrence of copulation were closely synchronized during the ovarian cycle and peaked around ovulation Female facial skin coloration triggers male's selective behavior	Wild; subjective color scores Free‐ranging; behavior experiment with color‐manipulated digital images of faces	46 47
		Facial luminance decreases between the pre‐conceptive month to the pregnancy period	Free‐ranging; digital photography; species‐specific visual models	48
		No evidence that female facial color is an indicator of age, dominance rank, parity or health	Free‐ranging; digital photography; species‐specific visual models	49
	Mandrill (*Mandrillus sphinx*)	The increase in redness of the sexual skin on the face and genitalia of males is related to social rank	Semi free‐ranging; color charts; color scores	50
		Females show preference for brightly colored males, independent of dominance rank	Semi free‐ranging; color charts; color scores	20
		Males may use red coloration to facilitate the assessment of dominance and subordination	Semi free‐ranging; color charts; color scores	19
		Color is not related to female rank or quality but may be a signal of reproductive quality	Semi free‐ranging; digital photography; objective measure of color	51
		Male facial redness is likely to reflects an honest signal of androgen status, competitive ability and willingness to engage in fights	Semi free‐ranging; digital photography; objective measure of color	52
		Red coloration is unrelated to parasitism and hematological parameters in male and females	Semi free‐ranging; digital photography; objective measure of color	53
		No relationship between red color and glucocorticoid levels	Semi free‐ranging; digital photography; objective measure of color	54
		Facial color increases with fecal androgen concentrations across females	Semi free‐ranging; digital photography; objective measure of color	55
	Drill (*Mandrillus leucophaeus*)	Male coloration indicates rank status.	Semi free‐ranging; digital photography; objective measure of color	18
	Baboon (*Papio sp*.)	Skin color is not related to menstrual cycle but is influenced by parity	Captive; color charts; color scores	56
		Skin color is not related to the timing of ovulation but may contain information about female parity	Wild; digital photography; objective measure of color	32
		Skin color is uninformative concerning the intracycle probability of fertility	Semi free‐ranging; digital photography; objective measure of color	57
	Gelada (*Theropithecus gelada*)	Red chest is a signal of one male unit holding status	Wild; digital photography; objective measure of color	17
	Black‐and‐white snub‐nosed monkey (*Rhinopithecus bieti*)	Lip redness increases with age and in the mating season	Semi‐provisioned; digital photography; objective measure of color	33
	Vervet monkey (*Cercopithecus sp*.)	Genital coloration is associated with intermale agonism; regulates the behavior of male competitors and may facilitate the evolution of multimale social system Males with darker scrotal color dominate males with pale scrota	Free‐ranging and captive; subjective color scores Captive; color charts; color scores; color manipulation and behavior assessment	16 58
		Females pay attention to male coloration, but do not bias their interactions towards males solely based on coloration Scrotum color varies between species; However, color variation may function as an age‐related signal to all species. Color is also related to morphological features (canine and body length)	Captive; color charts; color scores; color manipulation and behavior assessment Free‐ranging and captive; digital photography; objective measure of color	59 60
	Sanje mangabey (*Cercocebus sanjei*)	Increase of skin luminance during ovulation period but not during gestation	Wild; subjective color scores	61
Mexico, Central and South America	Common marmoset (*Callithrix jacchus*)	Skin chroma and luminance varies during the weeks surrounding parturition	Captive; spectrometry; species‐specific visual models	62

**Figure 1:**
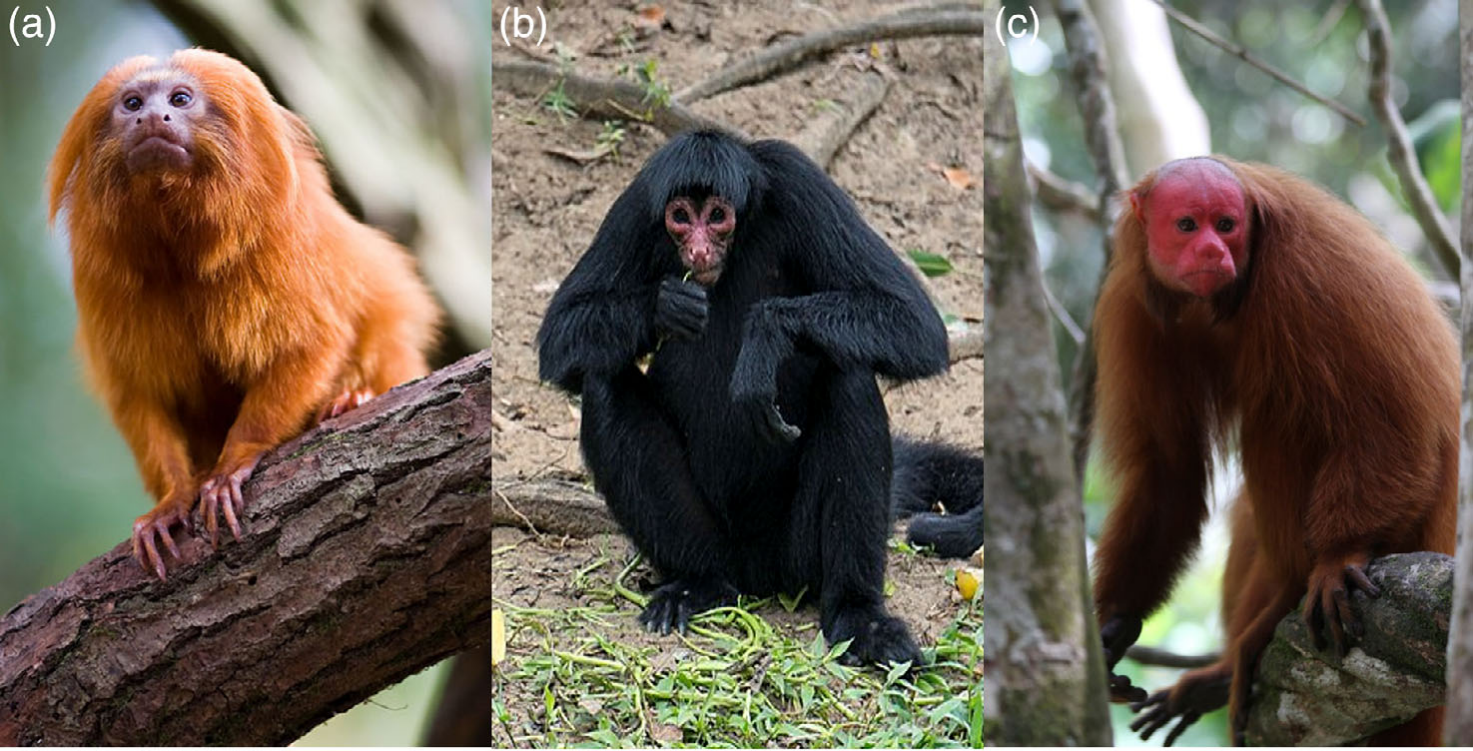
Examples of exposed facial skin in platyrrhine monkeys. Golden lion tamarins (*Leontopithecus rosalia*, a); red‐faced spider monkey (*Ateles paniscus*, b); and bald‐headed uakari (*Cacajao calvus*, c). Photo credits: Jeroen Kransen (a), Dan Sloan (b), and Marc Wisniak (c)

Facial skin that is distinctly reddish and/or exhibits large luminance contrast relative to surrounding pelage is also present in the other major radiation of haplorhine primates, the monkeys of Mexico, Central and South America (Parvorder Platyrrhini) (Figure 1). Intriguingly, a wide diversity of social systems occurs in platyrrhines, including polyandry and monogamy, begs investigating the impact of mating systems on signal evolution. However, comparative studies of platyrrhine coloration are almost absent in the literature. The only study investigating intraspecific skin color variation in platyrrhines demonstrates that female genital skin color (luminance and hue) varies across pregnancy and parturition in common marmosets (*Callithrix jacchus*).^62^ It is possible that this color variation is a nonadaptive by‐product of hormonal variation with no signaling purpose.^63^, ^64^ Alternatively skin color may be part of a suite of sensory cues associated with triggering preparation for male care,^65^ or female reproductive suppression.^66^ Although speculative, this work calls attention to the potential for intraspecific skin color signals in platyrrhine monkeys and highlights the need and potential for color signal studies within this clade.

In addition to color variation, the extent of exposed skin among the monkeys of the Americas ranges from the most extensive seen in the order (bald uakaris [*Cacajao calvus*]) to a complete absence of exposed skin (e.g. moustached tamarins [*Saguinus mystax*]). The increased surface area of exposed skin in African and Asian monkeys has been hypothesized to enhance communication based on skin color signals.^31^, ^67^ However, causal explanations are only supported if they are both explicable and predictable—that is, they demonstrate regularity and independence in their evolution in response to similar selective conditions.^68^ The platyrrhines provide an independent test of the hypothesis that the amount of exposed facial and genital skin is evolutionarily plastic, and when present, used in socio‐sexual communication. To be convergent with catarrhines, we would expect increased facial skin exposure to have evolved independently in platyrrhine species that use colorful skin signals.

## 3 EVOLUTION OF SKIN COLOR AND EXPOSURE IN PLATYRRHINE MONKEYS: A CHARACTER MAPPING APPROACH

To motivate future studies of visual signals and their role in communication among platyrrhine primates, we here provide the first analysis of exposed facial skin, variation, distribution, and evolution in Platyrrhini. Concordantly, we aim to uncover patterns underlying red, conspicuous skin. Because a dataset of color‐calibrated images across the platyrrhines is not yet available, our analysis is color‐subjective. We analyzed two to five forward‐facing images found in a current encyclopedia of living primate species^69^ and from online image libraries (Table S1). We quantified facial skin exposure and color for 96 platyrrhines, and for comparative purposes, 28 catarrhines, 7 strepsirrhines, 1 tarsiiform, and 13 nonprimate outgroups. Coding of skin color and exposure was done independently by L.A.A.M. and G.D. In case of any discrepancy, we recoded the images together to reach consensus. We did not include *Homo sapiens* in our analyses because of high intraspecific phenotypic variation and numerous derived features.

**Figure 2:**
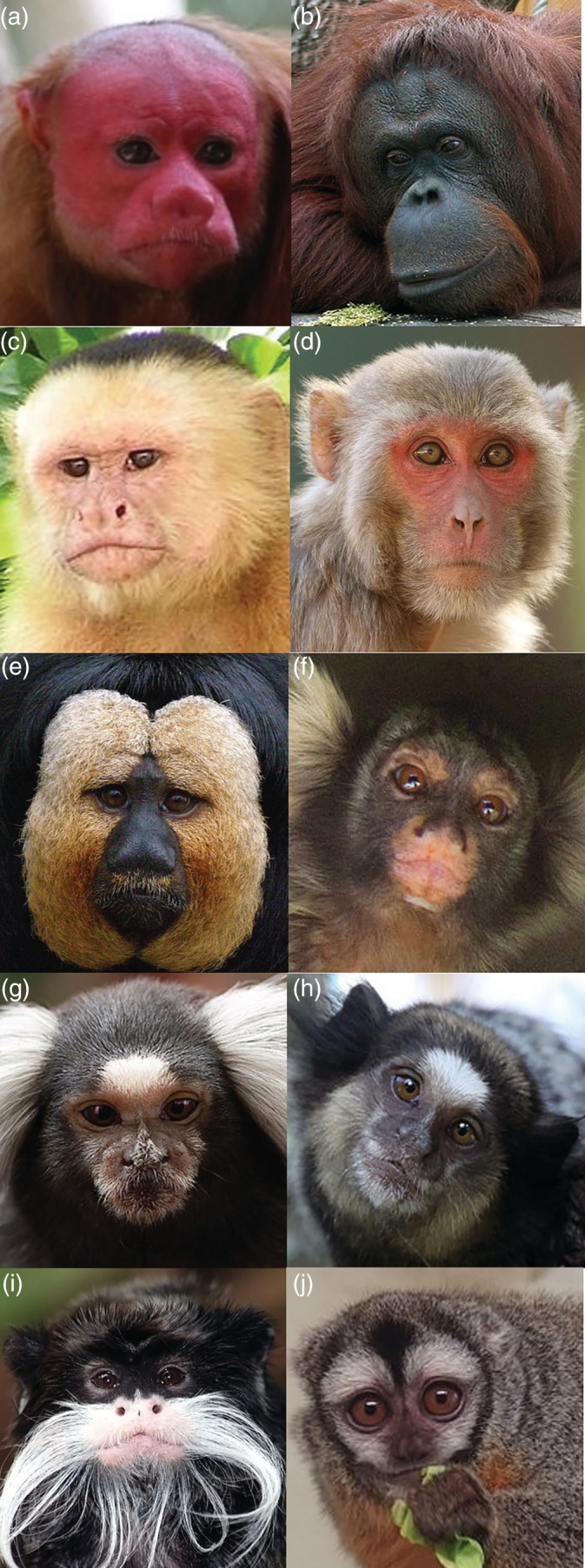
Examples of categories of exposed skin in primates. Completely exposed face (cheeks, nose, eyes, forehead), a, b); exposed skin around the eyes, nose, and mouth, c, d); exposed skin around the nose and eyes; or exposed skin around the nose and mouth, e, f); exposed skin around eyes, g, h); exposed skin around the nostrils, i, j). Photo credits: Marc Wisniak (a), Zyance (b), David M. Jensen (c), David V. Raju (d), Frank Wouters (e), Fabio Manfredini (f), Leszek Leszczynski (g), Halley Pacheco de Oliveira (h), Brocken Inaglory (i), and Jik jik (j)

**Figure 3:**
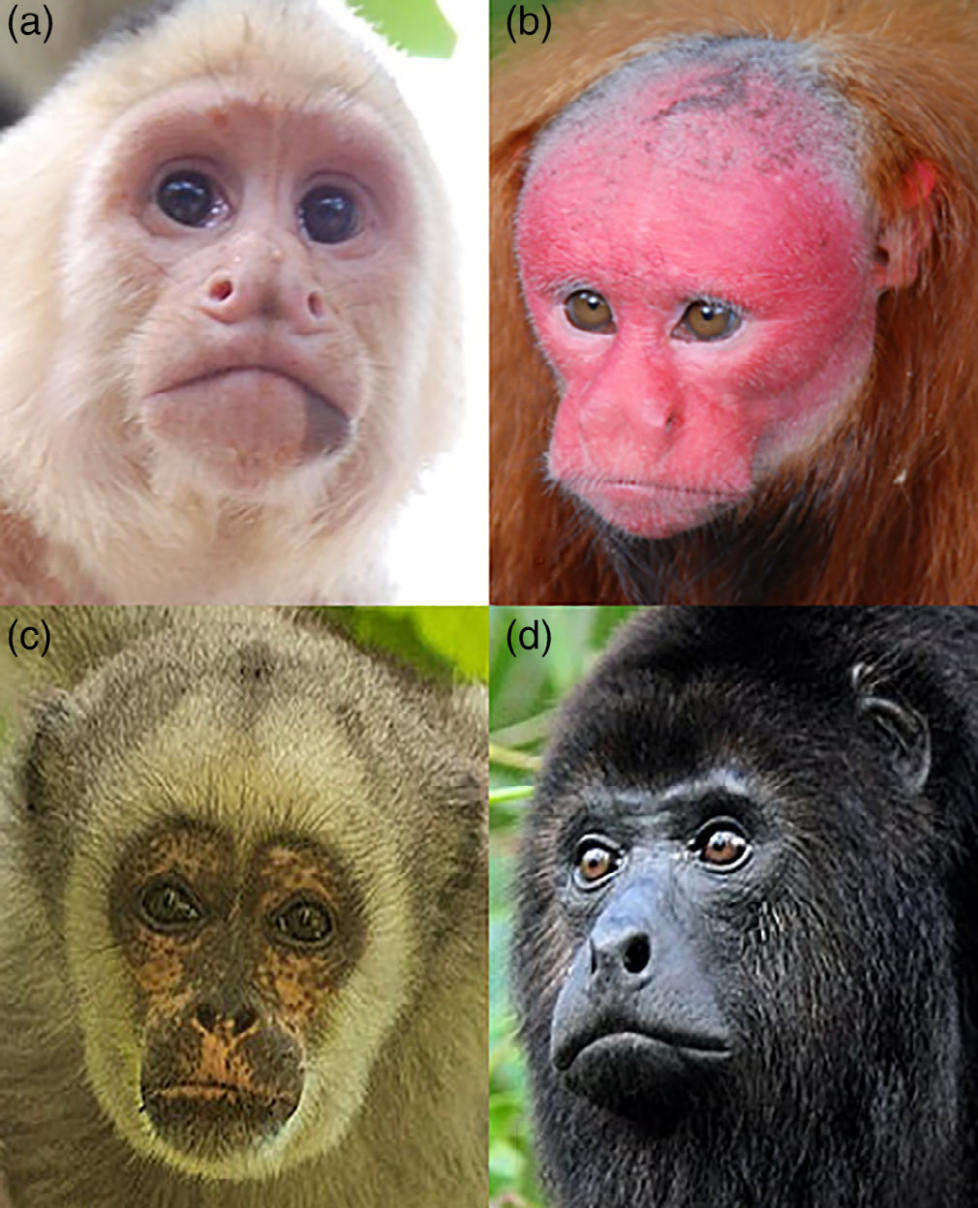
Examples of categories of skin color in primates. Depigmented (*Cebus capucinus*), a); hypervasculated (*Cacajao calvus*), b); mottled (*Brachyteles hypoxanthus*), c); hyperpigmented (*Alouatta pigra*), d). Photo credits: Steven G. Johnson (a), Kevin O'Connel (b), Peter Schoen (c), and Dave Johnson (d)

**Figure 4:**
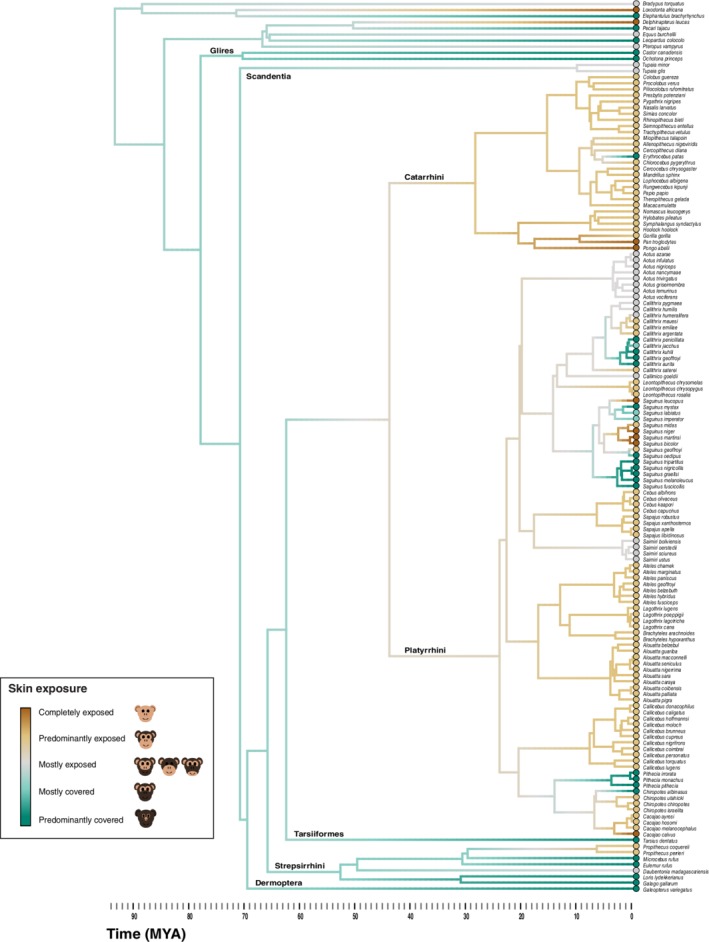
Ancestral state reconstruction of skin exposure visualized on a phylogeny of primates using Maximum Likelihood under the Ornstein‐Uhlenbeck (OU) model. The phylogenetic tree was redrawn from^70^ and adapted to include 96 platyrrhines, along with 28 catarrhines, 7 strepsirrhines, 1 tarsiiform, and 13 nonprimate groups to reconstruct ancestral types. The color map represents observed and reconstructed ancestral states for skin exposure ranging from a completely exposed face (brown) to only exposed skin on the nose (green)

**Figure 5:**
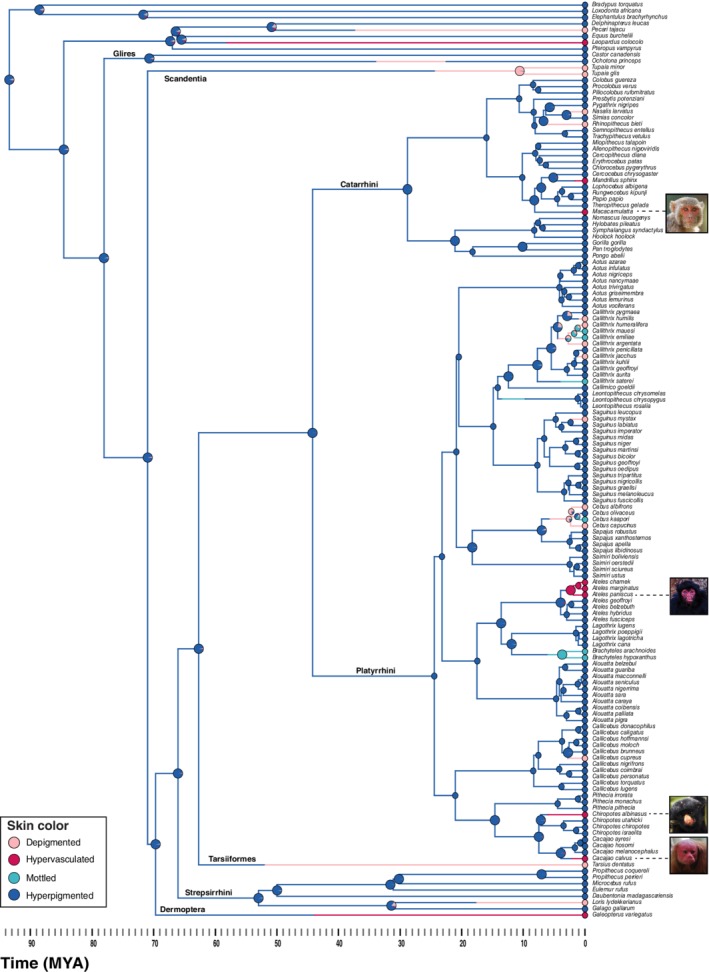
Ancestral state reconstruction of skin color visualized on a phylogeny of primates using 1,000 stochastic character maps under the Equal Rates (ER) model. The phylogenetic tree was redrawn from^70^ and adapted to include 96 platyrrhines, along with 28 catarrhines, 7 strepsirrhines, 1 tarsiiform, and 13 nonprimate groups to reconstruct ancestral types. Branch colors represent posterior probability densities of the skin color states along the edges and pie charts show the relative probabilities of each state at the internal nodes. Pink indicates depigmented skin, red indicates hypervasculated skin, light blue indicates mottled skin, and dark blue indicates hyperpigmented skin. Images of hypervasculated skin are shown on the right of the species names. Photo credits: Marc Wisniak (*Cacajao calvus*), Valdir Hobus (*Chiropotes albinasus*), Kitty Terwolbeck (*Ateles paniscus*) and David Raju (*Macaca mulatta*)

We created five categories to code patterns of increasing surface area of exposed facial skin: (a) completely exposed skin (cheeks, nose, eyes, forehead [Figure 2a,b]); (b) exposed skin around the eyes, nose and mouth [Figure 2c,d]; (c) exposed skin around the nose and eyes, or exposed skin around the nose and mouth [Figure 2e,f]; (d) exposed skin around eyes [Figure 2g,h]; and (e) exposed skin around the nostrils [Figure 2i,j]. We coded for skin color using established categories^29^: (a) depigmented (white skin [Figure 3a]); (b) hypervascularized (red skin [Figure 3b]); (c) mottled (depigmented skin with very small pigmented patches [Figure 3c]); and (d) hyperpigmented (dark skin [Figure 3d]). We then performed an ancestral state reconstruction by mapping the facial exposure (Figure 4) and color traces (Figure 5) on a current phylogenetic tree (auto‐correlated rates, soft‐bounded constraints).^70^, ^71^

The ancestral character states for skin exposure (continuous trait) were simulated using the Maximum Likelihood method. We tested three models of evolution: Brownian Motion (BM), Ornstein‐Uhlenbeck (OU), and Early Burst (EB), before selecting the best‐fitting model (OU). Skin exposure evolution was plotted using the phytools contMap function in R.^72^ Because the evolutionary history of monkeys in the Americas is still debated, we repeated our analyses across multiple potential phylogenies (Figures S1 and S2).^73^, ^74^ We inferred the evolutionary history of skin color (discrete trait) using a stochastic mapping approach implemented in the R phytools package.^72^ The ancestral states at each node were estimated under three basic models: equal rates (ER), all rates different (ARD), and symmetrical transition rate (SYM). The best model fitting (ER) was selected and 1,000 character histories were simulated across the phylogeny using the phytools make.simmap function in R version 3.5.2.^75^

Our results indicate that exposure of facial skin is an evolutionarily plastic trait, with shifts from relatively hairy to relatively exposed skin and vice versa being relatively common among platyrrhines (Figure 4). The last common ancestor (LCA) of catarrhines and platyrrhines is reconstructed to have predominantly exposed skin, that is, exposed skin around the eyes, mouth, and nose. Many species of platyrrhines possess a lesser amount of exposed skin than catarrhines. Yet, there are radiations of platyrrhines with facial skin that is largely to entirely exposed (Figure 4). Increases in exposed facial skin appear to have evolved independently in platyrrhines at least five times. We see this trait in the genus *Saguinus*, and among the Atelidae family, with interesting variation within the genus *Cacajao*. There is also considerable exposed skin within the genera *Cebus* and *Sapajus*, and the callitrichids, including some but not all species of *Callithrix*, some *Saguinus*, and all examined members of the genus *Leontopithecus* (Figure 4). Interestingly, some lineages including the genus *Pithecia* and the white‐nosed saki (*Chiropotes albinasus*) seem to have experienced re‐covering of the face in hair. Faces covered by hair are reconstructed as ancestral for strepsirrhines, although at least one genus (including nine species) *Propithecus*, has exposed facial skin.

Turning to chroma, we find a conspicuous reddish patch is present on the nose of the (seemingly inappropriately named) white‐nosed saki (*Chiropotes albinasus*), and for at least four species (bald uakaris [*Cacajao calvus*], red‐faced spider monkeys [*Ateles paniscus*], black‐faced spider monkey [*Ateles chamek*], and white‐cheeked spider monkey [*Ateles marginatus*]) the facial skin is predominantly red (Figure 5). Both bald uakaris and red‐faced spider monkeys are also among those with the most exposed facial skin, consistent with the hypothesis that the evolution of exposed faces and red signals are correlated.^31^ However, it is important to note that while this relationship occurs, many species with completely or predominantly exposed skin do not have red pigmentation, highlighting the presence of considerable variation (e.g., *Saguinus bicolor*; Figures 4 and 5).

Our results are robust to phylogenetic uncertainty, that is, we come to similar conclusions when the models are run with different phylogenies (Figures S3 and S4). Overall, we demonstrate that there is extensive variation among platyrrhines in the presence of exposed facial skin, and high potential for reddish color signals. We suggest this group offers great promise for studies that mirror the research programs that have been undertaken to examine skin signal evolution in other taxa.

**Figure 6:**
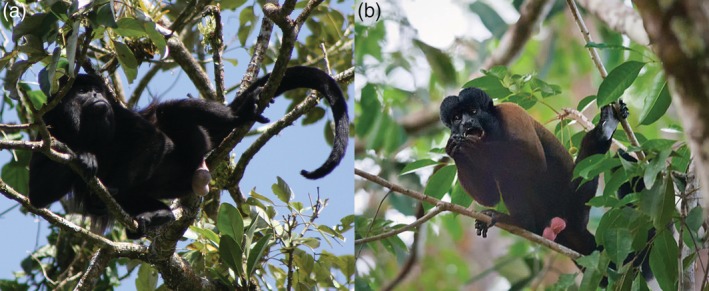
Conspicuous scrotum relative to body pelage in mantled howler monkey (*Alouatta palliata*, a) and bearded saki (*Chiropotes satanas*, b). Photo credits: Scott Robinson (a) and Allan Hopkins (b)

The analyses we present here consider only facial skin, as the diagnostic images of faces are more readily available for many species. However, like many African and Asian primates, the genital skin of at least some Central and South American monkeys is exposed, visible, and of high visual (luminance) contrast to body pelage (Figure 6). In some species of catarrhines, color is expressed on both the face and genitals, and this color variation is often correlated (e.g., rhesus macaques^35^; drills^18^). However, other species only exhibit conspicuous color on the genitals (e.g. vervet monkeys^16^). Variation in the conspicuity of genital skin among platyrrhines suggests that there may be convergent evolution on such traits, and that studies of inter‐ and intra‐specific variation in genital color alongside studies of face color are likely to be fruitful.

## 4 COLOR VISION AND OPSIN GENE DIVERSITY

**Figure 7:**
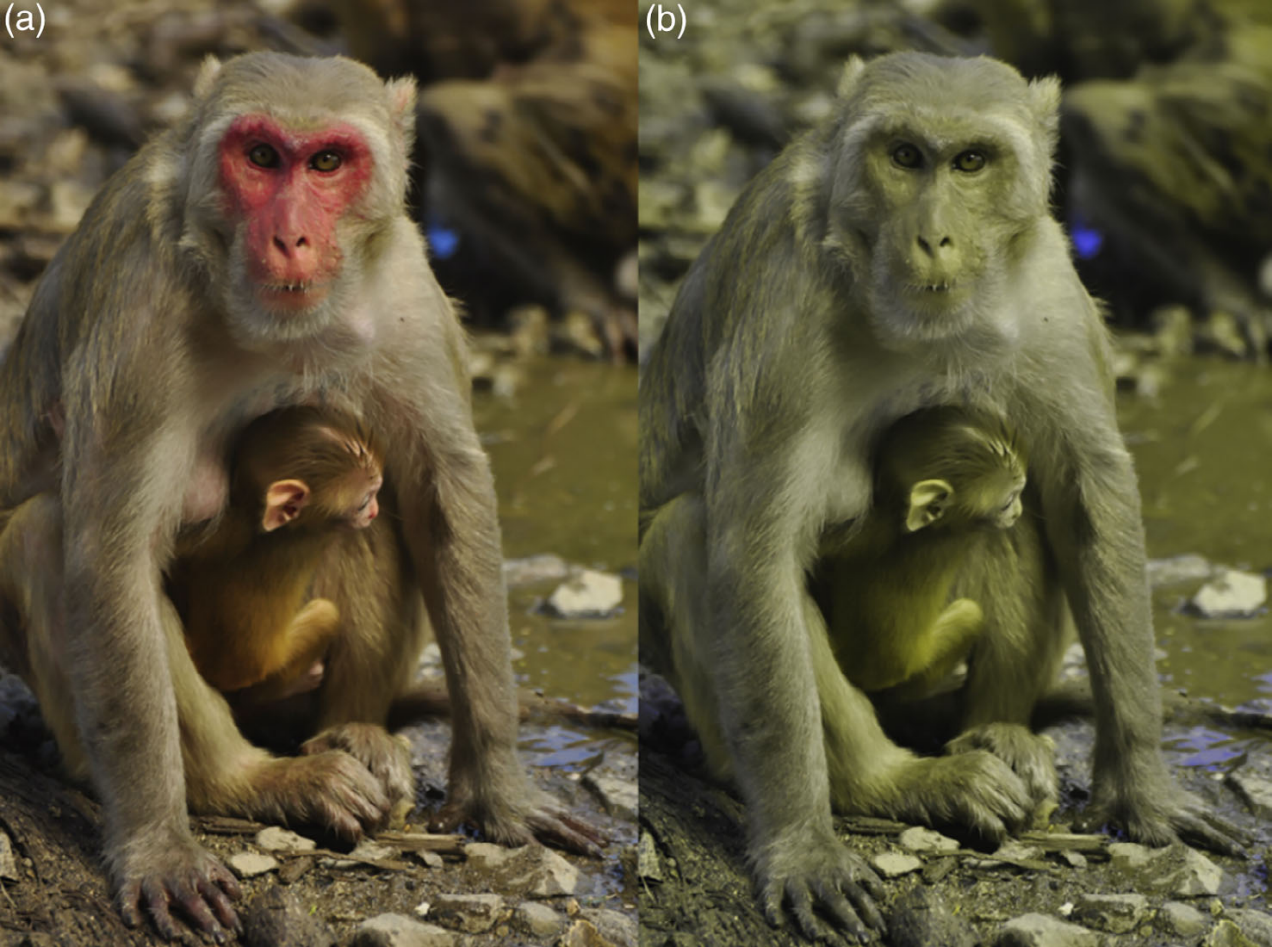
Simulated appearance of a female rhesus macaque (*Macaca mulatta*; Cayo Santiago) for: a trichromatic observer (a); a dichromatic observer (b).^80^ Peak cone sensitivity values on simulations of trichromatic Rhesus macaque vision (S cone = 420 nm; M cone = 530 nm; L cone = 560 nm) and a protanomalous dichromatic type (S cone = 420 nm; M cone = 530 nm).^45^ Photo by ADM

Characteristics of the color vision system are of critical importance to perception and interpretation of visual signals.^76^ Intriguingly, while catarrhine and platyrrhine primates share many aspects of their visual systems, there are important differences in their abilities to perceive color. Catarrhine primates have a uniform ability to discriminate among long‐wavelength hues (shades of red, orange, yellow, green) due to the duplication and divergence of an ancestral *OPN1LW* opsin gene.^45^, ^77^, ^78^, ^79^ Among platyrrhines, color vision is considerably more variable. Due to a uni‐locus *OPN1LW* polymorphism, all males and homozygous females have dichromatic (red‐green colorblind) vision, while a subset of females are heterozygous and have trichromatic color vision. This variation has important consequences for the perception of natural stimuli, including socially relevant scenes.^45^ To illustrate this impact of color vision type, we present an image of a female macaque with reddish facial skin as simulated for a trichromatic conspecific, and a hypothetical dichromatic observer (Figure 7). Accordingly, concomitant with studies of skin color variation in platyrrhines should be an assessment of species‐specific color vision, and development of species‐specific and individual‐specific models of skin conspicuity.^21^, ^35^, ^43^, ^48^, ^49^ Given the wide range of mating systems seen in platyrrhines, this group provides an unprecedented opportunity to ask questions about the role of skin chroma and luminance in attracting mates and competing with same‐sex conspecifics when communication may be occurring between individuals with different color perception systems.

With few exceptions^81^, ^82^ the entire body of work on platyrrhine color vision has assessed color vision variation through the lens of potential foraging adaptations. Although color vision has been linked to food detection and feeding efficiencies,^83^, ^84^, ^85^, ^86^ without an assessment of possible roles of color in socio‐sexual signaling, it is challenging to thoroughly assess the relative importance of diet in influencing color vision evolution. The sole study along this vein reveals exceptionally high frequencies of opsin polymorphism in uakari monkeys, thus presumably increasing the number of trichromatic females in the population; the high opsin diversity of this species might be linked to sexual selection and mate choice.^81^ Evaluating the diversity of opsin genes in other species with potential reddish signals, such as the red‐faced spider monkeys and the white‐nosed saki, would provide an independent test of this hypothesis.

## 5 NEW HORIZONS TO PURSUE AND CHALLENGES TO OVERCOME

Studying skin chroma and luminance variation in platyrrhines, alongside other understudied taxa (strepsirrhines, hylobatids), will add to the wealth of information now available for catarrhines and enable us to answer new questions about the variation and evolution of primate color signals. For example, this framework will promote tests of potential convergence in the evolution of color signals among distantly related species, and improve our ability to assess the socio‐sexual, ecological, and perceptual variables that shape the emergence, persistence, and form of different traits. Research questions could include the role of skin signals in male–male competition, mate choice, color signals as potential condition indicators, and the heritability of color traits, and their relationship to fitness. We suggest that studies in these areas should aim to: (a) document skin signal variation, (b) understand the genetic and physiological mechanisms involved in the production of skin color, (c) understand the relationship to behavior and/or biologically important traits such as ovulation, and (d) test these signals experimentally back to live animal receivers. The impressive array of work done on catarrhine primates, for which numerous viable methods have been developed (Table 1), provides excellent templates for such studies.

We hypothesize that luminance and/or chromatic variation is involved in socio‐sexual communication for at least some species of platyrrhine primates. Specifically, we predict that color variation of facial or genital skin plays a role in communication for species with exposed faces and genitals. If socio‐sexual communication among females along the red‐green channel is important, then those taxa are predicted to have greater numbers of opsin alleles and higher instances of trichromacy. By genotyping the *OPN1LW* opsins of platyrrhine primates with reddish, exposed faces, along with closely related species without red faces or patches, the hypothesis that red and visible facial skin is associated with increased diversity of opsin alleles in platyrrhines can be tested. It is of particular interest to note that among monkeys in the Americas, females may have a private color channel due to the sex‐linked nature of color vision variation in platyrrhines. A private channel is a signaling channel that only a subset of potential receivers can see. This has been suggested, for example, for bird UV signaling, which is visible to other birds, but not visible to mammalian predators.^87^ This terminology is also used commonly in the multimodal signaling literature, where signals expressed in different sensory channels are potentially available to different types of receivers.^88^ Redness may plausibly be a female–female specific signal of competitive ability, fertility and/or dominance, although some female observers may be excluded. If platyrrhine species are found to have reddish skin signals, and if there is evidence of trichromatic advantage in fitness, this may point to a role of heterozygote advantage in the socio‐sexual domain as a mechanism of balancing selection acting in these species. Such studies are therefore poised to make important contribution to our understanding of the diversity of evolutionary processes maintaining genetic variation.

In studies of the evolution of platyrrhine color vision, we urge a more holistic approach that integrates the role of natural selection in foraging and predator detection strategies with pressures linked to sexual selection and socio‐sexual communication. For example, species that are less reliant on cryptically colored foods, for which dichromacy is advantageous,^83^, ^89^ may have higher rates of opsin polymorphism, and greater opportunity for the evolution of reddish signals in social and sexual contexts. This scenario has been hypothesized to be a possible explanation for the highly polymorphic opsins of uakaris,^81^ but has not been systematically examined in any species. Similarly, if luminance is a salient and important channel for visual signals, selective pressure to communicate may impact balancing selection acting on opsin genes by favoring dichromatic phenotypes, as trichromatic color vision may corrupt perception of the luminance channel, which may in turn impact foraging ecology.^76^ Howler monkeys (*Alouatta* sp.), whose routine trichromacy has evolved independently from that of catarrhines, and monochromatic owl monkeys (*Aotus* sp.) may provide unique test cases that generate new insight into primates color vision evolution.^86^, ^90^ Finally, we note that it is important to explicitly evaluate female–female signaling, male–male signaling, female‐to‐male signaling, and male‐to‐female signaling separately in platyrrhines, given the sex‐linked nature of color vision variation.

Challenges to studying skin color signals in platyrrhines include: (a) difficulties in measuring coloration of small, highly arboreal primates; (b) finding appropriate populations for testing attention to signals through experimental image and/or model presentation; and (c) collecting biological samples with appropriate sampling of wild populations to assess opsin gene diversity within and between species. Sampling wild populations is crucial, as captive populations can have low sample sizes and decreased genetic heterozygosity, yet this is increasingly difficult given shrinking primate populations across Central and South America and Mexico. Possible solutions for each of these issues include: (a) innovating methods in color‐calibrated digital photography^91^ for low‐light conditions, or research designs that capitalize on opportunities to capture images of primates when they are relatively still and well lit, perhaps near canopy gaps; (b) integrating field experiments on platforms^92^ or study of captive or semi‐captive populations holds considerable promise^93^; (c) relying on DNA from noninvasively collected fecal samples, rather than blood, hair, or tissue, to increase the feasibility of population‐level sampling. Fortunately, study of opsin genes from fecal DNA is well established, alleviating need to capture and release wild monkeys.^94^, ^95^, ^96^ Careful and comprehensive sampling of wild populations is possible, if time consuming.^97^, ^98^

Finally, although we have focused on skin color and intraspecific communication, we would be remiss if we failed to highlight the potential of interspecific pelage and skin color variation to contribute to key evolutionary processes. For example, species boundaries may be enhanced via conspicuous character displacement among callitrichid and titi monkey lineages that have radiated widely in short time periods.^99^, ^100^, ^101^ Sensory ecology is at the heart of evolutionary anthropology—the major differences between the haplorhines and the strepsirrhines, and similarly between catarrhines and platyrrhines, are in the ways in which they receive and process information from their environment. A more comprehensive study of primate visual communication, to include understudied major radiations, will enhance our understanding of the evolution of our improved color vision and of social communication, key elements of what it means to be a primate.

## Supporting information


**Figure S1** Ancestral state reconstruction of skin exposure visualized on an alternate phylogeny of the Order Primates^73^ using Maximum Likelihood under the Ornstein‐Uhlenbeck (OU) model. The color map represents observed and reconstructed ancestral states for skin exposure ranging from completely exposed face (brown) to only exposed skin on the nose (green).

**Figure S2** Ancestral state reconstruction of skin exposure visualized on a Bayesian inference of primate phylogeny reconstructed by the 10kTrees Project.^74^ Ancestral states were inferred using Maximum Likelihood under the Ornstein‐Uhlenbeck (OU) model. The color map represents observed and reconstructed ancestral states for skin exposure ranging from completely exposed face (brown) to only exposed skin on the nose (green).

**Figure S3** Ancestral state reconstruction of skin color visualized on a Bayesian inference of primate phylogeny reconstructed by the 10kTrees Project.^74^ Ancestral states were inferred using 1,000 stochastic character maps under the Equal Rates (ER) model. Branch colors represent posterior probability densities of the skin color states along the edges and pie charts show the relative probabilities of each state at the internal nodes. Pink indicates depigmented skin, red indicates hypervasculated skin, light blue indicates mottled skin, and dark blue indicates hyperpigmented skin.

**Figure S4** Ancestral state reconstruction of skin color visualized on an alternate phylogeny of the Order Primates^73^ using 1,000 stochastic character maps under the Equal Rates (ER) model. Branch colors represent posterior probability densities of the skin color states along the edges and pie charts show the relative probabilities of each state at the internal nodes. Pink indicates depigmented skin, red indicates hypervasculated skin, light blue indicates mottled skin, and dark blue indicates hyperpigmented skin.

**Table S1 Additional sources of images for platyrrhine skin assessment.** Most coding was done by consulting a current encyclopedia of living primate species.^69^ Here we list additional image sources used to code skin exposure and color for species included in our analysis.
