# Supplementary material for: Platyrrhine color signals: New horizons to pursue
Source: Evol Anthropol. 2019 Oct 14;28(5):236–48. doi: 10.1002/evan.21798 (PMC6865018; doi:10.1002/evan.21798)
Supplement: Supplementary file 2 — Figure S2 Ancestral state reconstruction of skin exposure visualized on a Bayesian inference of primate phylogeny reconstructed by the 10kTrees Project.74 Ancestral states were inferred using Maximum Likelihood under the Ornstein‐Uhlenbeck (OU) model. The color map represents observed and reconstructed ancestral states for skin exposure ranging from completely exposed face (brown) to only exposed skin on the nose (green). [file EVAN-28-236-s002.pdf]

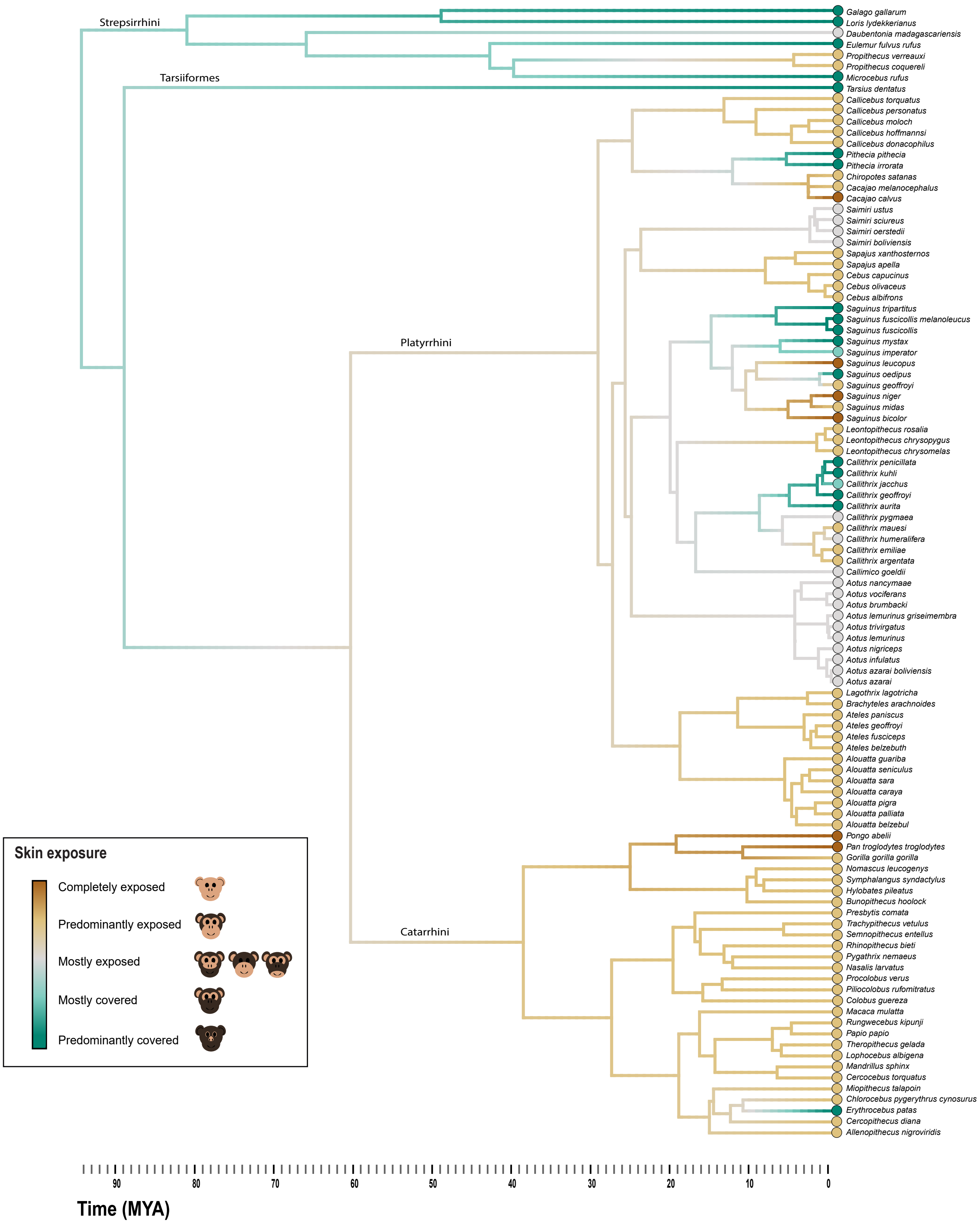

Figure S2 Ancestral state reconstruction of skin exposure visualized on a Bayesian inference of primate phylogeny reconstructed by the 10kTrees Project[74]. Ancestral states were inferred using Maximum Likelihood under the Ornstein-Uhlenbeck (OU) model. The color map represents observed and reconstructed ancestral states for skin exposure ranging from completely exposed face (brown) to only exposed skin on the nose (green).
