# Supplementary material for: Platyrrhine color signals: New horizons to pursue
Source: Evol Anthropol. 2019 Oct 14;28(5):236–48. doi: 10.1002/evan.21798 (PMC6865018; doi:10.1002/evan.21798)
Supplement: Supplementary file 3 — Figure S3 Ancestral state reconstruction of skin color visualized on a Bayesian inference of primate phylogeny reconstructed by the 10kTrees Project.74 Ancestral states were inferred using 1,000 stochastic character maps under the Equal Rates (ER) model. Branch colors represent posterior probability densities of the skin color states along the edges and pie charts show the relative probabilities of each state at the internal nodes. Pink indicates depigmented skin, red indicates hypervasculated skin, light blue indicates mottled skin, and dark blue indicates hyperpigmented skin. [file EVAN-28-236-s003.pdf]

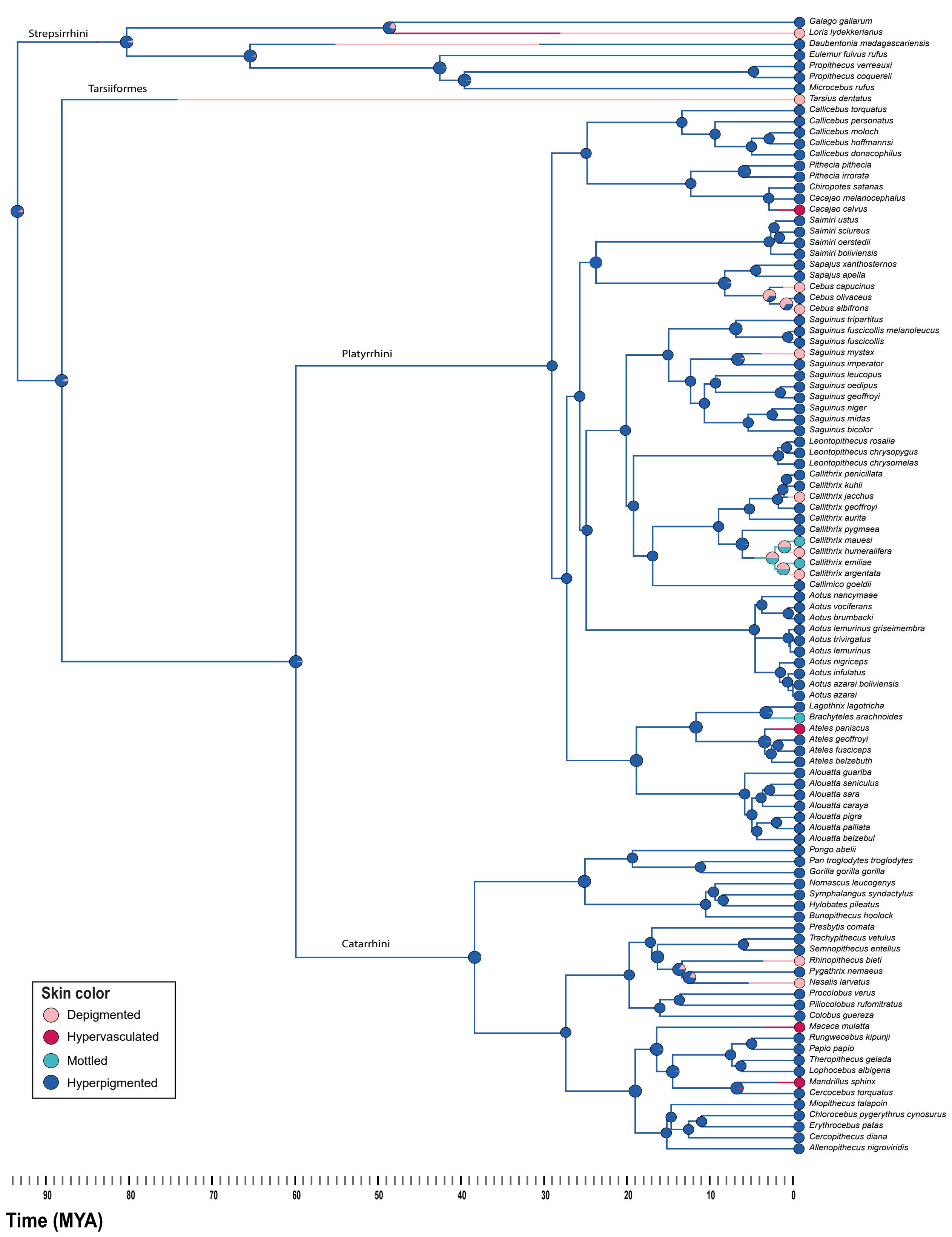

Figure S3 Ancestral state reconstruction of skin color visualized on a Bayesian inference of primate phylogeny reconstructed by the 10kTrees Project[74]. Ancestral states were inferred using 1000 stochastic character maps under the Equal Rates (ER) model. Branch colors represent posterior probability densities of the skin color states along the edges and pie charts show the relative probabilities of each state at the internal nodes. Pink indicates depigmented skin, red indicates hypervasculated skin, light blue indicates mottled skin, and dark blue indicates hyperpigmented skin.
