# Supplementary material for: Platyrrhine color signals: New horizons to pursue
Source: Evol Anthropol. 2019 Oct 14;28(5):236–48. doi: 10.1002/evan.21798 (PMC6865018; doi:10.1002/evan.21798)
Supplement: Supplementary file 4 — Figure S4 Ancestral state reconstruction of skin color visualized on an alternate phylogeny of the Order Primates73 using 1,000 stochastic character maps under the Equal Rates (ER) model. Branch colors represent posterior probability densities of the skin color states along the edges and pie charts show the relative probabilities of each state at the internal nodes. Pink indicates depigmented skin, red indicates hypervasculated skin, light blue indicates mottled skin, and dark blue indicates hyperpigmented skin. [file EVAN-28-236-s004.pdf]

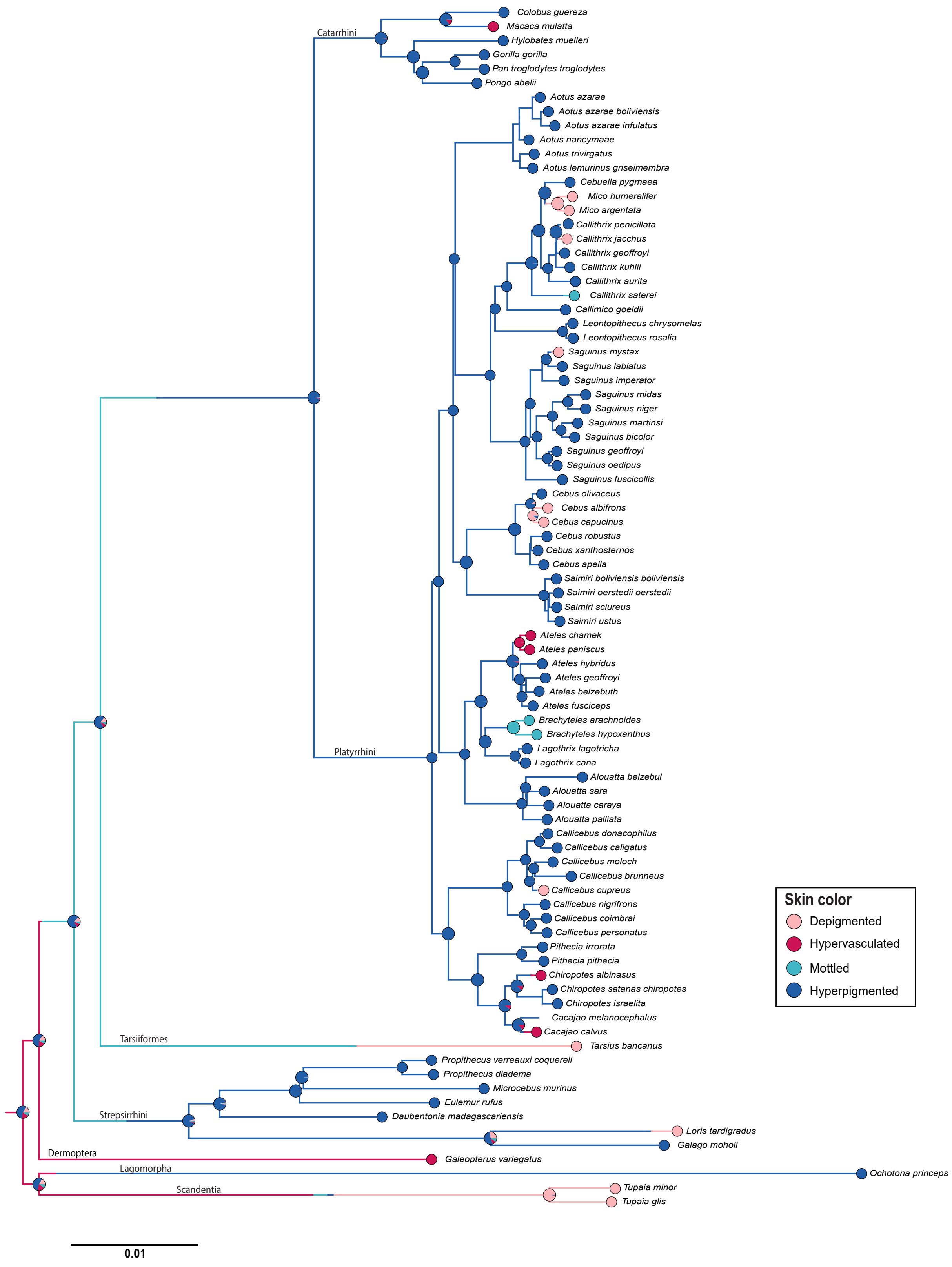

Figure S4 Ancestral state reconstruction of skin color visualized on an alternate phylogeny of the Order Primates[73] using 1000 stochastic character maps under the Equal Rates (ER) model. Branch colors represent posterior probability densities of the skin color states along the edges and pie charts show the relative probabilities of each state at the internal nodes. Pink indicates depigmented skin, red indicates hypervasculated skin, light blue indicates mottled skin, and dark blue indicates hyperpigmented skin.
