# Supplementary material for: Platyrrhine color signals: New horizons to pursue
Source: Evol Anthropol. 2019 Oct 14;28(5):236–48. doi: 10.1002/evan.21798 (PMC6865018; doi:10.1002/evan.21798)
Supplement: Supplementary file 5 — Table S1 Additional sources of images for platyrrhine skin assessment. Most coding was done by consulting a current encyclopedia of living primate species.69 Here we list additional image sources used to code skin exposure and color for species included in our analysis. [file EVAN-28-236-s005.pdf]

Table S1 Additional sources of images for platyrrhine skin assessment. Most coding was done by consulting a current encyclopedia of living primate species[42]. Here we list additional image sources used to code skin exposure and color for species included in our analysis.

**Species**	**Common Name**	**All the World's Primates Book**	**Digital Source of Image**
Galeopterus variegatus	Sunda flying lemur	✓	Arkive
Galago gallarum	Somali lesser Galago	✓	Arkive
Loris lydekkerianus	Gray slender loris	✓	Arkive
Daubentonia madagascariensis	Aye-aye	✓	Arkive
Eulemur rufus	Red brown lemur	✓	Arkive
Microcebus rufus	Brown mouse lemur	✓	Arkive
Propithecus perrieri	Perrier's sifaka	✓	Arkive
Propithecus coquereli	Coquerel's sifaka	✓	Arkive
Tarsius dentatus	Dian's tarsier	✓	Arkive
Cacajao calvus	Bald uakari	✓	Arkive
Cacajao melanocephalus	Golden-backed uakari	✓	Arkive
Cacajao hosomi	Neblina uakari	✓	ICMBio
Cacajao ayresi	Aracá uakari	✓	Arkive
Chiropotes israelita	Brown-backed bearded saki	✓	WikiSpecies
Chiropotes chiropotes	Bearded saki	✓	Arkive
Chiropotes utahicki	Uta Hick's bearded saki	✓	WikiSpecies
Chiropotes albinasus	White-nosed saki	✓	Arkive
Pithecia pithecia	White-faced saki	✓	Arkive
Pithecia monachus	Monk saki	✓	WikiSpecies
Pithecia irrorata	Gray's bald-faced saki	✓	Arkive
Callicebus lugens	Widow monkey	✓	WikiSpecies
Callicebus torquatus	Collared titi monkeuy	✓	IUCN
Callicebus personatus	Atlantic titi	✓	PrimateWisc
Callicebus coimbrai	Coimbra-Filho's titi monkey	✓	ICMBio
Callicebus nigrifrons	Black-fronted titi monkey	✓	ICMBio
Callicebus cupreus	Coppery titi monkey	✓	UCDavis
Callicebus brunneus	Brown titi monkey	✓	Arkive
Callicebus moloch	Red-bellied titi monkey	✓	IUCN

Callicebus hoffmannsi	Hoffmann's titi monkey	✓	Callicebus
Callicebus caligatus	Chestnut-bellied titi	✓	Callicebus
Callicebus donacophilus	Bolivian gray titi	✓	Arkive
Alouatta pigra	Yucatán black howler monkey	✓	Arkive
Alouatta palliata	Mantled howler monkey	✓	Arkive
Alouatta coibensis	Coiba island howler	✓	Arkive
Alouatta caraya	Black-and-gold howler monkey	✓	Arkive
Alouatta sara	Bolivian red howler monkey	✓	Wikimedia
Alouatta nigerrima	Black howler monkey	✓	iNaturalist
Alouatta seniculus	Red howler	✓	Arkive
Alouatta macconnelli	Guianan red howler monkey	✓	Wikimedia
Alouatta guariba	Brown howler monkey	✓	Arkive
Alouatta belzebul	Red-handed howler monkey	✓	Arkive
Brachyteles hypoxanthus	Northern muriqui	✓	Arkive
Brachyteles arachnoides	Southern muriqui	✓	Arkive
Lagothrix cana	Peruvian woolly monkey	✓	Arkive
Lagothrix lagotricha	Common woolly monkey	✓	Wikimedia
Lagothrix poeppigii	Red woolly monkey	✓	Arkive
Lagothrix lugens	Colombian woolly monkey	✓	Wikimedia
Ateles fusciceps	Brown-handed spider monkey	✓	Arkive
Ateles hybridus	Variegated spider monkey	✓	Arkive
Ateles belzebuth	White-bellied spider monkey	✓	Arkive
Ateles geoffroyi	Geoffroy's spider monkey	✓	Arkive
Ateles paniscus	Guiana spider monkey	✓	Arkive
Ateles marginatus	White-cheeked spider monkey	✓	Wikimedia
Ateles chamek	Black-faced black spider monkey	✓	Arkive
Saimiri ustus	Bare-eared squirrel monkey	✓	Wikimedia
Saimiri sciureus	Common squirrel monkey	✓	Arkive
Saimiri oerstedii	Black-crowned Central American squirrel monkey	✓	Arkive

Saimiri boliviensis	Black-headed squirrel monkey	✓	Arkive
Sapajus libidinosus	Bearded Capuchin	✓	ICMBio
Sapajus apella	Tufted capuchin	✓	Arkive
Sapajus xanthosternos	Buff-headed capuchin	✓	Arkive
Sapajus robustus	Crested capuchin	✓	Uwisconsin
Cebus capucinus	White-faced capuchin	✓	Arkive
Cebus kaapori	Kaapori capuchin	✓	Arkive
Cebus olivaceus	Wedge-capped capuchin	✓	Arkive
Cebus albifrons	White-fronted capuchin	✓	Arkive
Saguinus fuscicollis	Saddleback tamarin	✓	Arkive
Saguinus melanoleucus	White saddleback tamarin	✓	Wikimedia
Saguinus graellsi	Graell's black-mantle tamarin	✓	Wikimedia
Saguinus nigricollis	Black-mantle tamarin	✓	Wikimedia
Saguinus tripartitus	Golden-mantled saddleback tamarin	✓	Arkive
Saguinus oedipus	Cotton-tip tamarin	✓	Arkive
Saguinus geoffroyi	Geoffroy's tamarin	✓	Wikimedia
Saguinus bicolor	Pied tamarin	✓	Arkive
Saguinus martinsi	Martin' bare-face tamarin	✓	Mamiferos do Mundo
Saguinus niger	Black-handed tamarin	✓	Wikipedia
Saguinus midas	Red-handed tamarin	✓	Arkive
Saguinus imperator	Emperor tamarin	✓	Arkive
Saguinus labiatus	Red-chested moustached tamarin	✓	Wikipedia
Saguinus mystax	Moustached tamarin	✓	Arkive
Saguinus leucopus	Silvery-brown tamarin	✓	Arkive
Leontopithecus rosalia	Golden lion tamarin	✓	Arkive
Leontopithecus chrysopygus	Black lion tamarin	✓	Arkive
Leontopithecus chrysomelas	Golden-headed lion tamarin	✓	Arkive
Callimico goeldii	Goeldi's monkey	✓	Arkive
Callithrix saterei	Sateré marmoset	✓	Arkive

Callithrix aurita	Buffy-tufted-ear marmoset	✓	Arkive
Callithrix geoffroyi	Geoffroy's tufted ear marmoset	✓	Arkive
Callithrix kuhlii	Wied's marmoset	✓	Arkive
Callithrix jacchus	Common marmoset	✓	Arkive
Callithrix penicillata	Black-tufted marmoset	✓	Wikimedia
Callithrix argentata	Silvery marmoset	✓	Arkive
Callithrix emiliae	Emilia's marmoset	✓	Wikimedia
Callithrix mauesi	Maués marmoset	✓	Nature Picture Library
Callithrix humeralifera	Santarem marmoset	✓	Arkive
Callithrix humilis	Roosmalen's dwarf marmoset	✓	Arkive
Callithrix pygmaea	Pygmy marmoset	✓	Arkive
Aotus vociferans	Spix's night monkey	✓	Arkive
Aotus lemurinus	Colombian night monkey	✓	Arkive
Aotus griseimembra	Grey-handed night monkey	✓	Arkive
Aotus trivirgatus	Northern night monkey	✓	Arkive
Aotus nancymaae	Nancy Ma's night monkey	✓	Wikimedia
Aotus nigriceps	Black-headed night monkey	✓	Wikimedia
Aotus infulatus	Feline night monkey	✓	Wikimedia
Aotus azarae	Azara's night monkey	✓	Wikimedia
Pongo abelii	Sumatran orangutan	✓	Arkive
Pan troglodytes	Chimpanzee	✓	Arkive
Gorilla gorilla	Western gorilla	✓	Arkive
Hoolock hoolock	Western hoolock gibbon	✓	Arkive
Symphalangus syndactylus	Siamang	✓	Arkive
Hylobates pileatus	Capped gibbon	✓	Arkive
Nomascus leucogenys	Northern white-cheeked gibbon	✓	Arkive
Macaca mulatta	Rhesus macaque	✓	Arkive
Theropithecus gelada	Gelada	✓	Arkive
Papio papio	Guinea baboon	✓	Wikimedia

Rungwecebus kipunji	Kipunji	✓	Arkive
Lophocebus albigena	Grey-cheeked mangabey	✓	Arkive
Mandrillus sphinx	Mandrill	✓	Arkive
Cercocebus chrysogaster	Golden-bellied mangabey	✓	Arkive
Chlorocebus pygerythrus	Vervet monkey	✓	Arkive
Erythrocebus patas	Patas monkey	✓	Arkive
Cercopithecus diana	Diana monkey	✓	Arkive
Allenopithecus nigroviridis	Allen's swamp monkey	✓	Arkive
Miopithecus talapoin	Southern talapoin monkey	✓	Wikimedia
Trachypithecus vetulus	Purple-faced langur	✓	Arkive
Semnopithecus entellus	Northern plains gray langur	✓	Wikimedia
Rhinopithecus bieti	Black snub-nosed monkey	✓	Arkive
Simias concolor	Pig-tailed langur	✓	Arkive
Nasalis larvatus	Proboscis monkey	✓	Arkive
Pygathrix nigripes	Black-shanked douc langur	✓	Arkive
Presbytis potenziani	Mentawai langur	✓	Arkive
Piliocolobus rufomitratus	Eastern red colobus	✓	Arkive
Procolobus verus	Olive colobus	✓	Mamiferos do Mundo
Colobus guereza	Guezera	✓	Arkive
Ochotona princeps	American pika	✗	Wikipedia
Tupaia glis	Common treeshrew	✗	Wikimedia
Tupaia minor	Pygmy treeshrew	✗	Wikimedia
Castor canadensis	North American beaver	✗	IUCN
Elephantulus brachyrhynchus	Short-snouted elephant shrew	✗	IUCN
Pteropus vampyrus	Large flying fox	✗	Wikimedia
Leopardus colocolo	Pampas cat	✗	IUCN
Pecari tajacu	Collared peccary	✗	Nature Picture Library
Delphinapterus leucas	Beluga whale	✗	Nature Picture Library
Equus quagga	Plains zebra	✗	Wikimedia
Loxodonta africana	African bush elephant	✗	IUCN

Bradypus torquatus	Maned three-toed sloth	✕	IUCN
